# Gearbox Fault Diagnosis Based on MSCNN-LSTM-CBAM-SE

**DOI:** 10.3390/s24144682

**Published:** 2024-07-19

**Authors:** Chao He, Jarula Yasenjiang, Luhui Lv, Lihua Xu, Zhigang Lan

**Affiliations:** College of Intelligent Manufacturing and Industrial Modernization, Xinjiang University, Urumqi 830017, China; hcxd@stu.xju.edu.cn (C.H.); lvluhui_1998@stu.xju.edu.cn (L.L.); xlh97@stu.xju.edu.cn (L.X.); zg1667@stu.xju.edu.cn (Z.L.)

**Keywords:** gearbox, fault diagnosis, multi-scale feature extraction, long short-term memory networks, convolutional block attention module, squeeze-and-excitation

## Abstract

Ensuring the safety of mechanical equipment, gearbox fault diagnosis is crucial for the stable operation of the whole system. However, existing diagnostic methods still have limitations, such as the analysis of single-scale features and insufficient recognition of global temporal dependencies. To address these issues, this article proposes a new method for gearbox fault diagnosis based on MSCNN-LSTM-CBAM-SE. The output of the CBAM-SE module is deeply integrated with the multi-scale features from MSCNN and the temporal features from LSTM, constructing a comprehensive feature representation that provides richer and more precise information for fault diagnosis. The effectiveness of this method has been validated with two sets of gearbox datasets and through ablation studies on this model. Experimental results show that the proposed model achieves excellent performance in terms of accuracy and F1 score, among other metrics. Finally, a comparison with other relevant fault diagnosis methods further verifies the advantages of the proposed model. This research offers a new solution for accurate fault diagnosis of gearboxes.

## 1. Introduction

As a core transmission component in mechanical equipment, the gearbox plays a pivotal role in the operation of the system. However, gearboxes are prone to failures in harsh operating environments, which not only affect their own performance but can also lead to anomalies in other components of the mechanical system, potentially threatening the stability of the entire system [1,2]. Given this, research on gearbox fault detection and diagnosis becomes particularly important, as it is crucial for ensuring the normal operation of equipment and preventing major accidents [3].

Traditional fault diagnosis research involves time-domain, frequency-domain, and time-frequency-domain analysis to extract signal features. However, as vibration signals often exhibit nonlinear and non-stationary characteristics, the conventional Fourier transform-based signal processing methods have certain limitations. As mentioned by Jalayer et al. [4], the traditional methods may only learn similar features in the feature extraction process, and the learned features could have shift attributes, which may lead to misclassification. Saucedo-Dorantes et al. [5] noted that for signals with low computational demands and reaching a steady state, Fast Fourier Transform (FFT) provides an efficient analysis approach. However, when the signal characteristics deviate from stationarity, other signal processing methods become more suitable alternatives. It is worth noting that many current condition monitoring techniques often focus on identifying a single fault type in early fault detection, limiting their potential application in broader fault detection scenarios. Additionally, when the system’s characteristic frequencies are non-stationary, the FFT technique has certain drawbacks in signal analysis [5]. For example, spectral analysis implemented in high-frequency environments may not fully realize its fault identification potential. The challenge lies in the fact that, as the operating frequency (*fm*) increases, the existing accelerometer bandwidth sometimes struggles to cover the high-frequency vibration signals generated by critical mechanical structures (such as gearboxes), thereby limiting the accurate capture and analysis of these signals. Hasan et al. [6] proposed a method that extracts time-domain and frequency-domain features using the FFT and then uses a Support Vector Machine (SVM) classifier to classify these features for identifying gearbox faults. This approach achieved relatively high accuracy, but it mainly relied on a single feature (SD) to achieve high accuracy, which may not have fully utilized other features that could provide more discriminative information. On the other hand, traditional mechanical fault diagnosis methods require a significant amount of diagnostic expertise and signal processing computations in the feature extraction process. This process is both time-consuming and dependent on expert experience [7]. Furthermore, since their feature extraction and classification processes are designed independently, optimizing models asynchronously will consume a considerable amount of time and limit their diagnostic performance [8]. As mentioned above, traditional methods such as FFT have their advantages, and this paper explores gear fault diagnosis from another perspective—that of deep learning.

With the cross-development of information science and engineering technology, artificial intelligence technology has found widespread application in fault diagnosis. Compared to traditional fault diagnosis methods based on expert knowledge and signal preprocessing, deep learning-based approaches have significant advantages. Deep learning does not rely on manually designed feature extraction methods, but can automatically learn effective feature representations from large amounts of raw data, thus exhibiting stronger learning capabilities and generalization performance when faced with massive and complex data. This data-driven, end-to-end fault diagnosis model provides a new solution for improving the accuracy and robustness of fault detection and diagnosis, which is of great importance for enhancing equipment reliability and safety. Duan et al. [9] proposed a gear fault diagnosis method based on convolutional neural network (CNN) and particle swarm optimization-supported vector machine (PSO-SVM). This method first extracts the time-frequency feature statistics of the signal, then uses the convolutional neural network for secondary feature extraction of the time-frequency feature statistics, and finally employs the particle swarm optimization-supported vector machine for classification. The results show that this method has high accuracy and the shortest training time. Xia et al. [10] proposed a multi-sensor convolutional neural network-based diagnosis method that fuses multi-sensor signals for the compound fault diagnosis of gearboxes. These techniques have achieved significant results in the field of fault diagnosis. However, the relevant information of fault signals is often scattered across different scales, which cannot be ignored. Many current methods are still limited to single-scale feature analysis, which restricts the comprehensive capture of multi-scale fault characteristics. Therefore, developing effective means to accurately extract multi-scale features is crucial for improving classification accuracy. To this end, Lin et al. [11] proposed the FPN model, which enhances the recognition performance of multi-scale targets by integrating cross-layer features to capture information at different scales. Similarly, Cai et al. [12] employed multi-scale convolution kernels to deeply explore the multi-scale characteristics of the input data, further improving the model’s performance in feature extraction.

In the current research trends, the approach combining CNN and attention mechanisms has been widely applied in the field of fault diagnosis. Wang et al. [13] proposed a deep residual neural network algorithm for gearbox fault detection by integrating the convolutional block attention module (CBAM) into the ResNeXt50 network, which aims to enhance the extraction of image channel and spatial features. Xu et al. [14] further explored this concept and developed a compound fault diagnosis method for transmission based on multi-scale convolutional neural networks (MSCNN) and a channel-spatial attention module (CSAM). Furthermore, Cheng et al. [15] developed a hybrid transformer and CNN attention network (HTCAN) for stereo image super-resolution tasks, while Zhang et al. [16] proposed a two-stage deep learning network structure with a separated channel convolutional neural network combined with an attention network (SC-CNN-Attention) for ADHD patient recognition. These studies have demonstrated the powerful feature extraction and diagnostic capabilities of the CNN combined with the attention mechanism approach in various application domains.

This paper proposes the MSCNN-LSTM-CBAM-SE model for gearbox fault diagnosis, which integrates MSCNN and long short-term memory networks (LSTM). This combination not only effectively extracts and integrates multi-scale spatio-temporal features, but also learns the global dependencies in sequential data. Particularly, the introduction of CBAM-SE further enhances the model’s adaptability, enabling the model to automatically emphasize the features critical for diagnosis and suppress irrelevant information. The application of this comprehensive approach enables the MSCNN-LSTM-CBAM-SE model to comprehensively capture the complex features of the data, thus providing strong support for accurate gearbox fault diagnosis and improving the accuracy and robustness of the diagnosis.

The primary contributions presented in this paper are outlined below:

(1) Regarding gearbox fault diagnosis, this paper reviews the deep learning methods for gearbox fault diagnosis and proposes the MSCNN-LSTM-CBAM-SE fault diagnosis model. The model leverages MSCNN to deeply explore the time-frequency features of vibration signals, and utilizes LSTM to accurately capture the temporal dynamic characteristics of fault signals. Furthermore, the integration of the CBAM and the Squeeze-and-Excitation module (SE) significantly enhances the model’s perception capability of critical fault features, while effectively suppressing the interference of noise and irrelevant features. This integrated adaptive learning capability not only optimizes the feature fusion strategy, but also enhances the model’s generalization ability and robustness to the dynamic industrial environment. The efficient and accurate diagnostic capability of the MSCNN-LSTM-CBAM-SE model provides strong technical support for predictive maintenance and reduced equipment downtime, making it an important force in driving the development of industrial intelligence.
(2) By integrating the SE module into the CBAM module, the advantages of the channel attention mechanism and the inter-channel relationships are fully utilized, enhancing the model’s ability to capture critical fault features. The SE module can adaptively focus on the most important channel features in the vibration signals, highlighting the information crucial for fault diagnosis, while suppressing the influence of redundant and interfering features. This CBAM module, enhanced with the SE module, can not only capture the complementary information between channels and the details of their spatial correlation more comprehensively, but also significantly improves the model’s interpretability through the visualization of attention weights. Compared to the original CBAM module, the improved CBAM-SE module significantly reduces unnecessary computational burden, enabling the network to learn the complex fault patterns in the vibration signals more efficiently, and providing more reliable support for the final fault diagnosis.
(3) The proposed MSCNN-LSTM-CBAM-SE fault diagnosis model was compared with five other designed models: CNN1-CBAM-SE, CNN2-CBAM-SE, CNN1-LSTM-CBAM-SE, CNN2-LSTM-CBAM-SE, and MSCNN-LSTM-CBAM-SE (w/o BN). This study utilized the HUST gearbox dataset and the WT-Planetary gearbox dataset to conduct a comprehensive experimental validation of the proposed MSCNN-LSTM-CBAM-SE fault diagnosis model. The experimental results showed that compared to the other five deep learning-based diagnosis models, the proposed method achieved significant performance improvements in terms of accuracy, F1 score, and other metrics.
(4) Through ablation experiments, the performance of each module was compared. Additionally, comparisons were made with several other fault diagnosis methods, which demonstrated the advantages of the proposed model. This indicates that the designed diagnostic framework, which integrates multi-scale convolutional feature extraction, temporal feature modeling, and the integration of attention mechanisms, can more effectively capture the critical fault features embedded in the vibration signals, thereby significantly improving the accuracy and reliability of gearbox fault diagnosis.

The remainder of this paper is organized as follows: Section 2 provides a detailed introduction to the application of deep learning methods in gearbox fault diagnosis and the basic theory of LSTM and CBAM-SE. Section 3 introduces the MSCNN-LSTM-CBAM-SE structure model and its intelligent fault diagnosis method. In Section 4, the evaluation criteria and comparison methods are introduced, and experimental studies are conducted on two datasets to verify the feasibility and superiority of the proposed method. Ablation experiments on the proposed model and comparisons with other fault diagnosis methods are also performed. Section 5 discusses the conclusions, limitations, and future research directions of this work.

## 2. Related Work

### 2.1. The Application of Deep Learning Methods in Gearbox Fault Diagnosis

The remarkable achievements of deep learning across various industrial domains can be attributed to its exceptional ability to capture and learn complex features from massive datasets. This capability has been particularly evident in the field of gearbox fault diagnosis, where deep learning methods have emerged as a powerful tool. Faced with the complexity of vibration signal data, deep learning offers the ability to perform in-depth analysis, providing unprecedented intelligent diagnostics and decision-making support. This has been a driving force in the advancement of industrial smart monitoring and maintenance technologies.

Convolutional neural networks (CNNs), with their powerful feature extraction capabilities, can automatically identify fault-related patterns from vibration signals. Chen et al. [17] proposed a deep learning method based on CNNs for the identification and classification of gearbox faults. The researchers used vibration signals as input, and through in-depth analysis of the signals using CNNs, they achieved the diagnosis of different fault modes in the gearbox. The paper considered different combination patterns based on some basic fault conditions and used 20 different test cases, each containing 12 different basic condition pattern combinations. The vibration signals were preprocessed by time-domain statistics (such as standard deviation, skewness, and kurtosis), and in the frequency domain, they were decomposed into multiple frequency bands through Fast Fourier Transform (FFT), and the root-mean-square (RMS) value of each band was calculated to maintain the shape of the spectral peak energy. Yu et al. [18] proposed a new deep neural network model, called one-dimensional residual convolutional autoencoder (1-DRCAE), for directly learning and extracting features from vibration signals for gearbox fault diagnosis. This model uses unsupervised learning to extract features through a one-dimensional convolutional autoencoder and reconstructs the filtered signal through a deconvolution operation. Additionally, residual learning was adopted to improve the model’s feature learning capability on one-dimensional vibration signals. Saufi et al. [19] designed and developed a deep learning model based on a stacked sparse autoencoder (SSAE) to handle the limited data sample problem caused by sensor issues in gearbox fault diagnosis. The researchers proposed a time-frequency image pattern recognition-based fault diagnosis system that can achieve high-accuracy diagnostic results even with limited data samples. Shi et al. [20] proposed a deep learning method based on a bidirectional convolutional long short-term memory (BiConvLSTM) network to solve the fault diagnosis problem of planetary gearboxes. This method can automatically and simultaneously extract spatial and temporal features from vibration and speed measurement data to determine the type, location, and direction of gearbox faults. By integrating CNNs into long short-term memory (LSTM) networks, BiConvLSTM can learn the spatial correlations and temporal dependencies between different sensor signals without losing key fault-sensitive features. As can be seen from the above, deep learning (DL) fault diagnosis techniques have received widespread attention. DL algorithms can learn multilayer representations from input data through a deep architecture with multiple data processing units, where the output of the previous layer serves as the input to the subsequent layer, and each layer can learn higher-level data representations from the output of the previous layer. Therefore, DL architectures can automatically extract multiple complex features from input data without human intervention. In DL methods, the signals captured by sensors can be directly used as model inputs, and the diagnosis results can be directly obtained after training the deep network [21,22]. Currently, there are several commonly seen types of deep learning models:

(1) Auto-encoder (AE): AE consists of an encoder and a decoder. AE encodes the raw input vector using an artificial neural network (ANN), and the decoder reconstructs the encoded vector to be as close as possible to the original input vector. In other words, the encoded vector can represent the features of the original input vector. Li et al. [23] proposed a deep transfer non-negative constrained sparse autoencoder, which leverages the advantages of deep learning and transfer learning to solve diagnostic problems with a small amount of labeled data. Zhang et al. [24] used an ensemble deep autoencoding network for fault diagnosis, which combines a sparse deep autoencoder, a denoising deep autoencoder, and a compressive deep autoencoder. This method can effectively handle redundant information, noise damage, and signal interference, and has shown high diagnostic performance on three data sets.
(2) Deep belief network (DBN): A DBN is composed of Restricted Boltzmann Machines (RBMs), which are generative models that learn to represent the input data as a probabilistic model and can be used to generate new data. Yang et al. [25] proposed an intelligent fault diagnosis method for wind turbine planetary gearboxes based on an optimized DBN. The method combines the optimized DBN and Sigmoid units with pulse feature extraction, achieving a high fault diagnosis accuracy. Hu et al. [26] proposed an improved deep belief network and applied it to rolling bearing fault diagnosis. The method utilizes the forward training part, which is composed of RBMs, to learn the hidden features of the vibration data. Then, through weight allocation, the reverse generative part generates samples to expand the dataset. Compared to other methods, the improved DBN demonstrates superior performance in bearing fault diagnosis.
(3) Convolutional neural network (CNN): CNN are similar to multi-layer perceptrons, but they use local connectivity and weight sharing to reduce the number of network parameters. Huang et al. [27] proposed an intelligent fault diagnosis method for wind turbine gearboxes based on wavelet packet decomposition and CNNs. The method first decomposes the vibration signals using wavelet packet decomposition, and then feeds the signal components into a hierarchical CNN for fault identification. He et al. [28] proposed a fault detection method based on dilated convolutional neural networks. The network takes two-dimensional data as input and, compared to traditional CNNs, the dilated convolutional neural network can maintain a larger receptive field, accelerate the monitoring speed, and is more suitable for real-time fault monitoring. The effectiveness of the method was verified using a wind turbine gearbox as an example. Jiang et al. [29] used multi-scale convolution to identify gearbox faults, which significantly improved the recognition accuracy compared to single-scale approaches. Reference [30] proposed a multi-channel convolutional neural network that takes vibration signal images as input for wind turbine fault diagnosis. Experiments have proven its ability to diagnose common faults.
(4) Recurrent neural network (RNN): A RNN can retain information from previous time steps in a sequence to a certain degree, and are widely used for modeling time series signals. However, RNNs suffer from the gradient vanishing or an exploding problem during backpropagation. Long short-term memory (LSTM) networks can effectively address this issue. Lu et al. [31] used raw vibration signals as the dataset and employed LSTM and deep neural networks to solve the problem of early fault diagnosis. Yin et al. [32] proposed a wind turbine gearbox fault diagnosis method based on an LSTM neural network with an optimized cosine loss (Cos-LSTM). The introduction of the cosine loss mitigates the influence of signal intensity, thereby improving the diagnosis accuracy. The effectiveness of the method was validated using fault vibration data collected from a gearbox fault diagnosis experimental platform.
(5) Generative adversarial networks (GANs): GANs generate satisfactory outputs through the adversarial training of a generator and a discriminator. Zhang et al. [33] proposed a GAN-based wind turbine gearbox fault diagnosis model to address the class imbalance problem. Huang et al. [34] presented an improved label noise-robust auxiliary classifier generative adversarial network for wind turbine gearbox bearing fault diagnosis. Through adversarial learning, the method generates diverse fault samples and exhibits high robustness to noisy labels. Compared to other methods, this approach achieves higher diagnosis accuracy under the constraints of limited data and noisy labels.

Overall, deep learning techniques have achieved significant breakthroughs in gearbox fault diagnosis, demonstrating great application potential. This paper adopts the MSCNN-LSTM-CBAM-SE method for gearbox fault diagnosis. By integrating multi-scale feature extraction, temporal analysis, attention mechanisms, and feature selection, this approach overcomes the limitations of traditional models under varying operating conditions. The MSCNN module is able to capture multi-scale time-frequency features, providing the model with rich fault-related information. The LSTM network effectively processes time series data and captures the dynamic changes in fault signals. The CBAM-SE module further enhances the model’s perception of critical fault features while suppressing the interference of noise and redundant information. This integrated approach enables the model to not only adapt to different data distributions but also achieve effective fault diagnosis in new or changing operating conditions, significantly improving the model’s generalization capability and diagnostic accuracy.

### 2.2. Long Short-Term Memory Network

**Figure 1:**
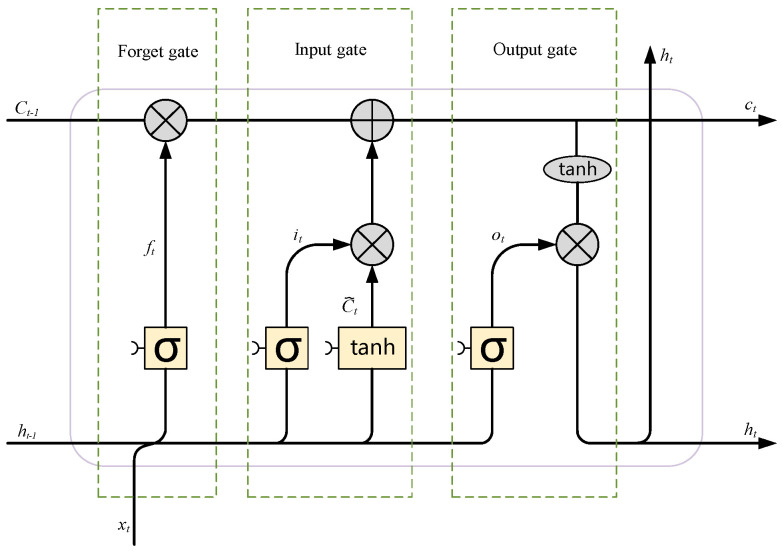
Internal structure of an LSTM with a forget gate.

Traditional recurrent neural networks (RNNs) [35] possess memory capabilities but tend to encounter gradient explosion and vanishing gradient issues when processing longer sequences, thereby failing to effectively learn long-term dependencies in input data. To address this problem, Hochreiter introduced the LSTM network [36]. As a variant of RNNs, LSTM networks have the ability to selectively retain valuable short-term and long-term information and achieve long-term memory. LSTM networks consist of LSTM cells, which introduce the concept of “gates” to enhance the memory capacity of the units. Following this pioneering work, researchers have made various improvements and extensions to LSTM, including variants without forget gates, with forget gates, and with peephole connections. Given that the forget gate LSTM is the most widely applied LSTM unit, this study chooses it as the basic unit structure and makes improvements upon it. The internal structure of such a unit is shown in Figure 1.

As shown in Figure 1, the mathematical expression for the LSTM unit is

(1) $$f_{t}=\sigma (W_{fh}h_{t-1}+U_{fx}x_{t}+b_{f})$$

(2) $$i_{t}=\sigma (W_{ih}h_{t-1}+U_{ix}x_{t}+b_{i})$$

(3) $$o_{t}=\sigma (W_{oh}h_{t-1}+U_{ox}x_{t}+b_{o})$$

(4) $${\tilde{c}}_{t}=\tan h(W_{\tilde{c}h}h_{t-1}+U_{\tilde{c}x}x_{t}+b_{\tilde{c}})$$

(5) $$c_{t}=f_{t}\cdot c_{t-1}+i_{t}\cdot {\tilde{c}}_{t}$$

(6) $$h_{t}=o_{t}\cdot \tan h(c_{t})$$

where $f_{t}$, $i_{t}$, and $o_{t}$ represent the forget gate, input gate, and output gate at time *t*, respectively; $c_{t}$, $h_{t}$, and $x_{t}$ denote the cell state, hidden state, and input unit at time *t*, respectively; W and U are the weights for the hidden state and input unit.

The forget gate has the function of filtering information, allowing it to decide which data should be excluded from the cell state. If $f_{t}$ = 1, it means that the information in the cell state will be completely retained; conversely, if $f_{t}$ = 0, it indicates that the information will be entirely removed.

### 2.3. Convolutional Block Attention Module

**Figure 2:**
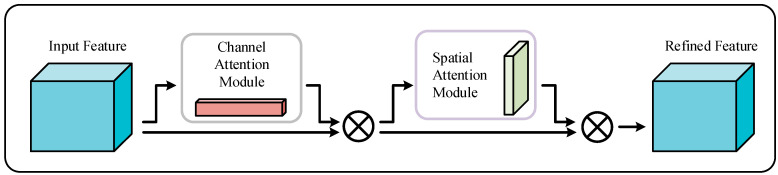
Structure of CBAM.

The convolutional block attention module (CBAM) is a convolutional neural network module based on the attention mechanism, designed to enhance the network’s capabilities in feature extraction and representation [37]. The structure of CBAM is shown in Figure 2, which includes a channel attention module (CAM) and a spatial attention module (SAM). The mathematical formula for the CBAM attention mechanism is as follows:

(7) $$F^{\prime }=M_{C}\left(F\right)⊗F$$

(8) $$F^{\prime \prime }=M_{S}\left(F^{\prime }\right)⊗F^{\prime }$$

where *F* belongs to $R^{C\times H\times W}$ as the input to the CBAM module, where *W* × *H* represents the feature dimensions of the data, and *C* is the number of channels. *M_C_*(*F*) belongs to $R^{C\times 1\times 1}$ as the output of the one-dimensional convolution of the CAM, which is also the input to SAM. *M_S_* ($F^{\prime }$) belongs to $R^{1\times H\times W}$ as the output of the SAM. $F^{\prime \prime }$ is the output of the CBAM module.

**Figure 3:**
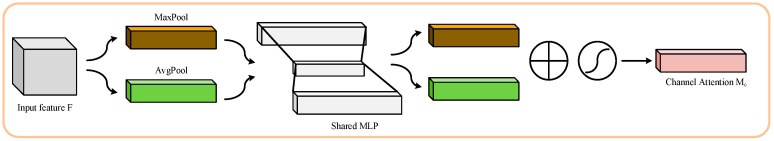
Architecture of the CAM.

The structure of the CAM is shown in Figure 3. It focuses more on the channel dimension of the input feature maps, aiming to learn the importance between channels, primarily through global average pooling and fully connected layers. Global average pooling allows for the averaging operation across each channel, thereby obtaining the global distribution information of the channels. The fully connected layers map this global distribution information to a lower-dimensional space, and the feature weight vector is obtained through an activation function.

The mathematical formula for the CAM attention mechanism is as follows:

(9) $$\begin{array}{c} M_{C}(F)=\sigma (MLP(AvgPool(F))+MLP(MaxPool(F)))\\ =\sigma (W_{1}(W_{0}(F_{avg}^{c}))+W_{1}(W_{0}(F_{\max}^{c}))) \end{array}$$

where $W_{0}\in R^{C/r\times C}$, $W_{1}\in R^{C\times C/r}$, σ represents the activation function.

**Figure 4:**
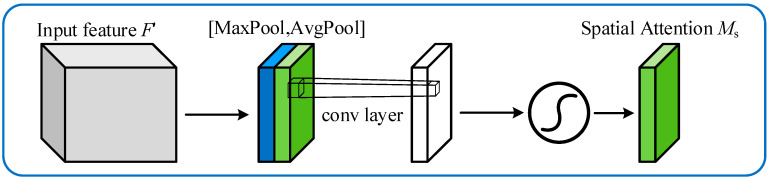
Architecture of the SAM.

The structure of the SAM is illustrated in Figure 4. It focuses more on the spatial dimension of the feature map, aiming to learn the relationships between spatial locations. This module includes a max pooling operation, followed by a convolution operation on the pooled features. Max pooling captures the most prominent feature at each spatial location, representing it in the feature map. Then, through a convolution operation, the feature map undergoes channel transformation and nonlinear mapping to further enhance the association between different spatial locations. This adaptive adjustment enhances the network’s focus on important features, improving feature discriminability and generalization performance. CBAM can be seamlessly integrated into various deep neural networks and has achieved notable performance improvements in tasks such as image classification, object detection, and image segmentation.

The mathematical formula for the SAM attention mechanism is as follows:

(10) $$\begin{array}{c} M_{S}\left(F\right)\left.=\sigma \left(\left[AvgPool\left(F\right);MaxPool\left(F\right)\right]\right)\right)\\ \left.\left.=\sigma \left(f\left(\left[\begin{array}{c} F_{avg}^{s};F_{\max}^{s} \end{array}\right]\right.\right.\right)\right) \end{array}$$

where f represents a convolutional layer with a 7 × 7 kernel.

This paper proposes an improvement to the existing CBAM approach by incorporating the Squeeze-and-Excitation (SE) module. The workflow is as follows:

(1) Computation of channel attention in the SE block: the input feature map is first passed through the SE module, which helps to emphasize important channel features and ignore less important ones.
(2) Global average pooling: global average pooling is used to compress the spatial information of each channel into a single value, forming a feature vector of length equal to the number of channels.
(3) Fully connected layer processing: The feature vector is processed through two fully connected layers. The first layer is typically used for dimensionality reduction to extract the most important features, while the second layer expands the dimensions back to the original channel count and uses a Sigmoid activation function to ensure the weights are between 0 and 1.
(4) Obtaining SE block channel attention weights: the weights obtained from the SE module are combined with the original channel attention weights of the CBAM module, which can enhance the model’s judgment of the importance of channels.
(5) Application of channel attention weights: the obtained channel attention weights are multiplied with the input feature map to strengthen the features of important channels and suppress the features of less important channels.
(6) CBAM’s spatial attention module: the channel attention feature map then enters the CBAM’s spatial attention module, where convolutional operations are used to learn the importance of spatial locations.
(7) Obtaining the final output: finally, the channel attention and spatial attention are multiplied to obtain the final feature map, which will contain strengthened important features and suppressed unimportant features.

**Figure 5:**
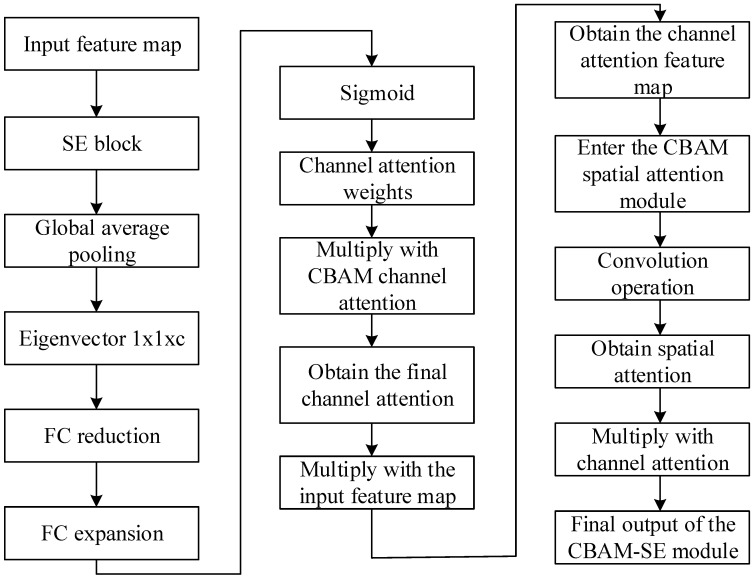
The principal flowchart of CBAM–SE.

The flowchart is shown in Figure 5 below.

In the MSCNN-LSTM-CBAM-SE gearbox fault diagnosis model, the CBAM-SE module plays a crucial role. Through the carefully designed CBAM-SE module, we not only inherit the channel attention and spatial attention mechanisms of CBAM, but also incorporate the deep feature selection capability of the SE module. This combination significantly enhances the model’s ability to capture subtle changes in vibration signals, enabling the model to more accurately identify and locate potential faults in the gearbox. The introduction of the CBAM-SE module makes the network more flexible and efficient in processing multi-scale and multi-dimensional features, effectively enhancing its perception and representation capabilities of fault characteristics.

## 3. Proposed Method

### 3.1. MSCNN-LSTM-CBAM-SE Structural Model

**Figure 6:**
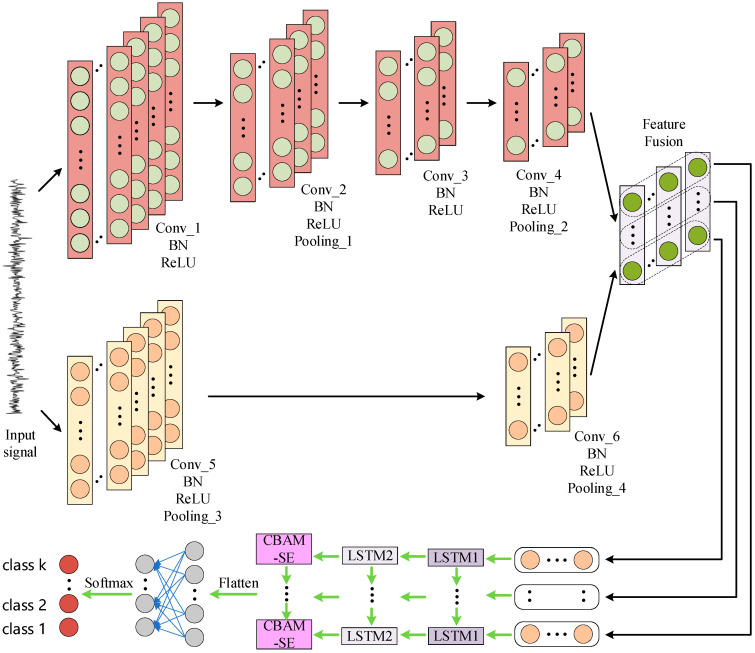
MSCNN–LSTM–CBAM–SE network structure.

MSCNN-LSTM-CBAM-SE is a hybrid neural network model specifically designed for processing and classifying vibration signal data. This model integrates a MSCNN and LSTM, along with the CBAM-SE, to effectively extract and utilize the features of time-domain signals. Figure 6 illustrates the structure of the MSCNN-LSTM-CBAM-SE model.

The method proposed in this paper enables neural networks to directly extract internal feature representations from normalized vibration signals to detect the health status of gearboxes. Prior to training, all samples are normalized using the Z-score method to unify the dimensions, facilitating subsequent computations. The input samples are original time-domain signals with a length of (1, 2048).

The feature extractor of the model consists of two parts: one is a parallel convolutional network using large kernels, and the other is a parallel convolutional network using small kernels. These two networks separately process the low-frequency and high-frequency information in the signal. In the network with large kernels, two convolutional layers with kernel sizes of 20 × 1 (Conv5 and Conv6) are used, which helps to capture the low-frequency components in the signal. In the network with small kernels, a series of 6 × 1 kernels (Conv1 to Conv4) are used to identify the high-frequency details in the signal. The outputs of the two networks are fused through element-wise multiplication, combining high and low-frequency information to enhance the expressiveness of the features. The fused features are then transposed to serve as input for the LSTM network.

Additionally, the model contains two LSTM layers (LSTM1 and LSTM2), which sequentially process the fused features. The LSTM network is capable of capturing long-term dependencies in time-series data, as the output of each time step affects the computation of the next time step. The hidden state of LSTM1 is used as the input to LSTM2. After the LSTM layers, the features pass through an improved CBAM-SE module, which is an attention mechanism that can adaptively adjust the importance of different features. Subsequently, the features are compressed through an adaptive average pooling layer, reducing the spatial dimension of the feature maps to 1. Finally, the pooled features are flattened and mapped to the target classes through a fully connected layer. The softmax function converts the output of the fully connected layer into a probability distribution, representing the likelihood of different classes. Ultimately, the class label with the highest probability is selected as the diagnostic result. In this way, the MSCNN-LSTM-CBAM-SE model can effectively process and classify the raw time-domain vibration signals, providing support for gearbox health monitoring and fault diagnosis.

It is worth noting that this paper introduces Batch Normalization (BN) layers into the CNN to enhance training stability and speed, reduce internal covariate shift, and prevent model overfitting. In this work, we particularly emphasize the importance of incorporating BN layers into CNN. This strategy not only helps in accelerating the convergence speed of the model and increasing the learning rate but also effectively mitigates the phenomenon of overfitting, thereby enhancing the model’s generalization ability. This is especially important for gearbox fault diagnosis, as accurate fault detection requires the model to maintain stable performance when faced with new, unseen data.

Specifically, MSCNN and LSTM, as feature extractors, exhibit the following significant advantages in gearbox fault diagnosis:

(1) Automatic feature learning: MSCNN and LSTM can automatically learn discriminative features from raw vibration signals without the need for manual design or selection of features. This is particularly important for complex gearbox fault patterns.
(2) Parameter optimization: CNN reduces the number of model parameters through the use of convolutional kernels with shared weights, which not only lowers the model’s complexity but also reduces the consumption of computational resources. In the MSCNN-LSTM-CBAM-SE model, the high-dimensional input vectors are effectively compressed through well-designed convolutional and pooling layers, further reducing the model’s parameters.
(3) Capturing global features: LSTM layers are capable of capturing long-term dependencies in time-series data, which is crucial for understanding the operational state of gearboxes and identifying potential fault patterns. By integrating LSTM layers after MSCNN, the model can synthesize local features and global contextual information, thereby improving the accuracy of fault diagnosis.
(4) Enhanced generalizability: The introduction of LSTM enables the model to not only learn local features but also mine global characteristics of vibration signals, significantly enhancing the model’s generalizability. The model can effectively perform fault diagnosis even when faced with different types of gearboxes or noise conditions.

In summary, by integrating multi-scale convolutional layers and long short-term memory networks, the MSCNN-LSTM-CBAM-SE model provides a powerful tool for gearbox fault diagnosis. It is capable of automatically extracting key features and handling various complex vibration signals while maintaining high accuracy. In this way, the model effectively supports maintenance decisions, allowing for the early prediction and prevention of potential equipment failures, thereby ensuring the stable operation and extending the service life of gearboxes.

### 3.2. Fault Diagnosis Method Based on MSCNN-LSTM-CBAM-SE

**Figure 7:**
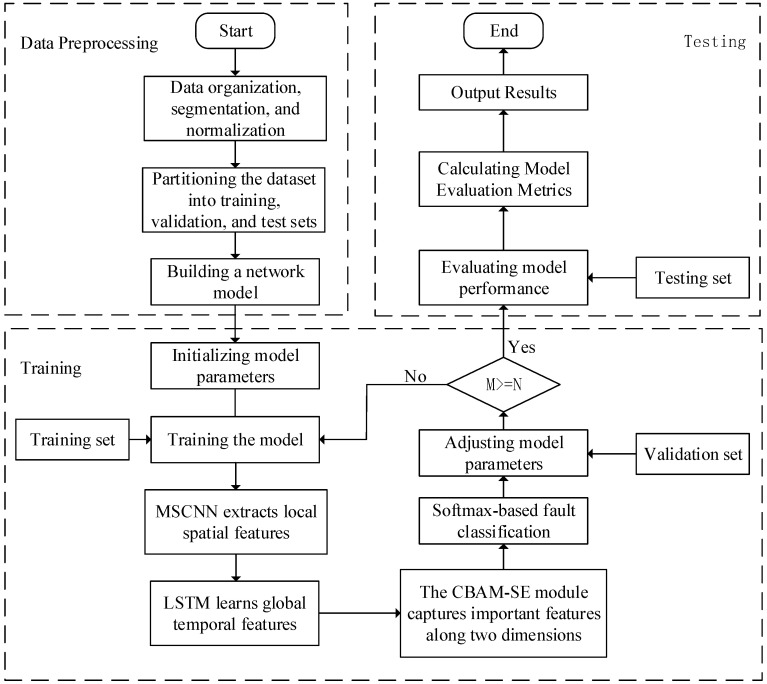
Intelligent fault diagnosis process.

This paper proposes an intelligent gearbox fault diagnosis process based on MSCNN-LSTM-CBAM-SE, which is outlined in Figure 7. It mainly consists of three steps: (1) data preprocessing; (2) training the MSCNN-LSTM-CBAM-SE model; (3) testing the model.

(1) Data preprocessing

In this study, the data preprocessing process followed these steps: First, by writing specific data loading functions, we read the raw data files from the data storage path and organized and classified them according to their categories. Then, using stratified sampling methods, we divided the organized dataset into training and testing sets, ensuring that the distribution ratio of each category in both sets remained consistent with the original dataset. This approach helps the model to better learn the features of each category. Additionally, we provided flexibility in data transformation, allowing for necessary preprocessing operations, such as normalization, to adapt to the model’s input requirements. Through this series of rigorous preprocessing steps, we provided a reliable data foundation for the training and evaluation of the model.

**Figure 8:**
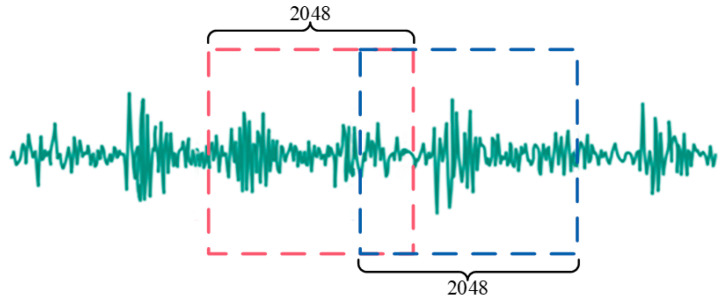
Illustration of overlapping sampling.

Additionally, this paper increased the number of samples through sliding window resampling, overlappingly reading vibration signals to enhance the robustness of the trained model. An illustrative diagram of the overlapping sampling with a sample length of 2048 is shown in Figure 8.

(2) Training the MSCNN-LSTM-CBAM-SE model

In multi-class fault diagnosis tasks, the error between the predicted values and the actual values is generally calculated using cross-entropy. Therefore, this paper also employs it to construct the objective function, as shown in the following expression:

(11) $$L=-\frac{1}{n}\sum_{i}(y_{i}\ln\mathrm{softmax}(z_{i}))$$

(12) $$\mathrm{softmax}(z_{i})=\frac{\exp(z_{i})}{\sum_{j=1}^{K}\exp(z_{j})}$$

where $y_{i}$ represents the true value, and $z_{i}$ denotes the output of the ith neuron in the last fully connected layer; softmax($z_{i}$) is the probability output of the ith neuron after passing through the softmax classifier; n represents the number of training samples; K denotes the number of categories.

The specific training process is as follows:

- Feature extraction and fusion: The normalized signals are input into two parallel convolutional networks (layer1 and layer2). Layer1 uses smaller convolutional kernels to capture high-frequency information, while layer2 uses larger convolutional kernels to capture low-frequency information. The features extracted by these two networks are fused through element-wise multiplication, forming a richer feature representation. The fused features are then fed into LSTM layers (LSTM1 and LSTM2), where the LSTM layers utilize their memory cell capabilities to extract temporal features, compensating for the convolutional networks’ shortcomings in capturing global features.
- Loss calculation and backpropagation: The fused features are weighted by the CBAM-SE module for spatial attention, highlighting important features and suppressing unimportant ones. Subsequently, the features are dimensionally reduced through an adaptive average pooling layer and mapped to the final output space through a fully connected layer. The difference between the model’s output and the true labels is measured by the cross-entropy loss function. The calculated loss value is propagated back through the network using the backpropagation algorithm to update the weights within the network.
- Iterative optimization: Through multiple iterations of training, the model parameters are continuously optimized until the performance of the model reaches a satisfactory level, or the preset number of iterations is achieved. In each iteration, the model calculates the output through forward propagation, then updates the parameters through backpropagation. In this way, the model gradually learns the patterns in the data, continuously improving its predictive performance throughout the training process.

(3) Testing the Model

Upon completing the training of the MSCNN-LSTM-CBAM-SE model, the diagnostic model will be deployed for fault classification, with test samples input into the model for validation.

## 4. Experimental Verification

### 4.1. Evaluation Criteria and Comparison Methods

As shown in Table 1, in the binary classification confusion matrix, there are four types of relationships between the predicted values and labels: True Positive, False Negative, True Negative, and False Positive. Based on the proportion of the number of samples in different relationships, four classification evaluation metrics are used to assess the model: accuracy, precision, recall, and F1 score. The calculation expressions are as follows:

(13) $$\mathrm{Accuracy}=\frac{TP+TN}{TP+TN+FP+FN}$$

(14) $$\mathrm{Precision}=\frac{TP}{TP+FP}$$

(15) $$\mathrm{Recall}=\frac{TP}{TP+FN}$$

(16) $$\mathrm{Fl}\;\mathrm{score}=\frac{2TP}{2TP+FP+FN}$$

To verify the performance of the proposed MSCNN-LSTM-CBAM-SE method, the following models were designed for comparative experiments:

(1) Four-layer small-kernel convolutional network: CNN1-CBAM-SE;
(2) Two-layer large-kernel convolutional network: CNN2-CBAM-SE;
(3) Combination network of CNN1 and two-layer LSTM: CNN1-LSTM-CBAM-SE;
(4) Combination network of CNN2 and two-layer LSTM: CNN2-LSTM-CBAM-SE;
(5) MSCNN-LSTM-CBAM-SE network without BN layer: MSCNN-LSTM-CBAM-SE (w/o BN);
(6) The method proposed in this paper: MSCNN-LSTM-CBAM-SE.

**Figure 9:**
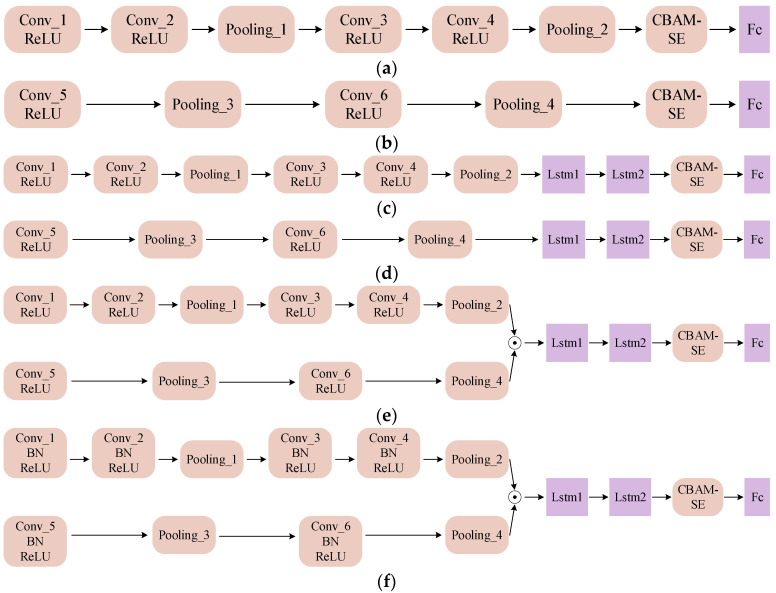
Main structures of the experimental models: (**a**) CNN1–CBAM–SE; (**b**) CNN2–CBAM–SE; (**c**) CNN1–LSTM–CBAM–SE; (**d**) CNN2–LSTM–CBAM–SE; (**e**) MSCNN–LSTM–CBAM–SE (w/o BN); (**f**) MSCNN–LSTM–CBAM–SE.

The parameter list for MSCNN-LSTM-CBAM-SE is shown in Table 2, and the main structures of the six models are illustrated in Figure 9. The networks containing LSTM layers are trained using the Adam algorithm. The networks that only have convolutional layers and no LSTM layers are trained using SGD. A total of 50 iterations were performed, with the iteration learning rate set to 0.0001, and the batch size during the entire training process was set to 64.

### 4.2. Experiment 1: Fault Diagnosis of Wind Turbine Planetary Gearbox

#### 4.2.1. Experimental Data Description

**Figure 10:**
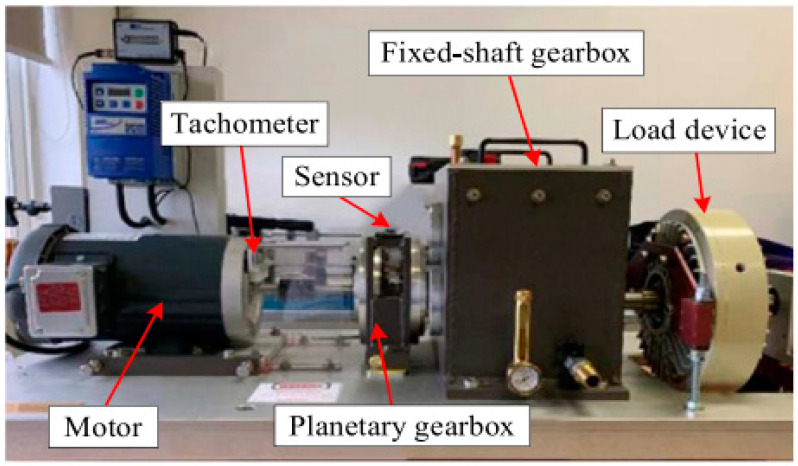
Wind power generator drivetrain testing setup.

**Figure 11:**
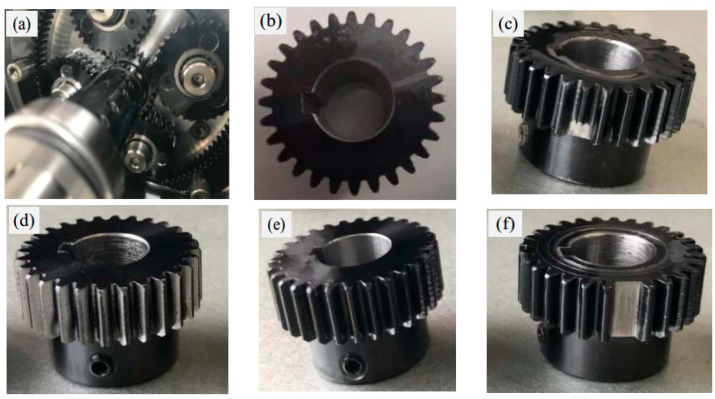
(**a**) Configuration of planetary gearbox internals; (**b**) healthy condition; (**c**) broken tooth; (**d**) worn tooth; (**e**) cracked tooth; (**f**) missing tooth.

The data acquisition test rig consists of a motor, a planetary gearbox, a fixed-axis gearbox, and a load device, as shown in Figure 10. The dataset has five health conditions and eight rotational speeds, also considering different installation effects. Among them, there are four planet gears revolving around a sun gear, as shown in Figure 11, with the five health conditions of the sun gear illustrated in Figure 11b–f. The vibration data were collected by a Sinocera CA-YD-1181 accelerometer, and the speed pulses were collected by an encoder, with all channels sampled at 48 kHz [38]. This dataset simultaneously collects two vibration signals in the x and y directions and the encoder data of the planetary gearbox input shaft, with eight speed conditions, ranging from 20 Hz to 55 Hz, considering each health condition. As in actual applications, the training data for the model include both historical data and real-time data, which need to be collected from different devices. Therefore, when conducting model experiments, device-related factors need to be considered. In this dataset, both disassembly and installation data were collected, covering the health conditions under eight operating conditions. Table 3 lists the detailed parameters of the planetary gearbox and the relationship between the two key fault-related frequencies and the input shaft frequency.

Some notes on this dataset:

(1) The scale of this dataset is massive, providing over 5 min of continuous recordings for each operating condition of the gearbox. In contrast to the widely used CWRU dataset, where signal lengths are typically only 10 s, this dataset has significantly extended the sample length. Past research has often set the sample length between 1000 to 2000 points. However, if the sample length is increased to 2000 points, even without overlapping sampling, each health condition can generate over 7000 samples. Such a sample size is more than sufficient for the training and optimization of deep learning networks.
(2) The vibration signals in the X and Y axes, as well as the encoder data of the planetary gearbox input shaft, were collected under stable conditions. This data collection was performed under eight different speed conditions, with each condition corresponding to a specific health state.
(3) In this dataset, device factors were considered, so the dataset includes all health conditions under eight operating conditions, and the disassembly and installation data were collected separately.

#### 4.2.2. Experimental Results and Analysis

The WT-Planetary gearbox dataset contains five states of gear health: broken tooth, healthy, missing tooth, crack, and wear [38]. For each state, there are two conditions related to the gear’s status: disassembled and installed. In the experiments, a set of 2048 data points in the X direction was selected as the dataset for each sample. Data for each installed condition at a speed of 20Hz were chosen, with 1000 samples extracted for each fault condition of the installation. The samples were divided into training and testing sets at an 8:2 ratio, meaning there were 800 samples for the training set and 200 samples for the testing set under each condition of gear health. Detailed information is presented in Table 4.

**Figure 12:**
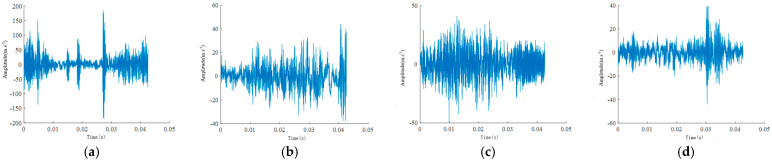

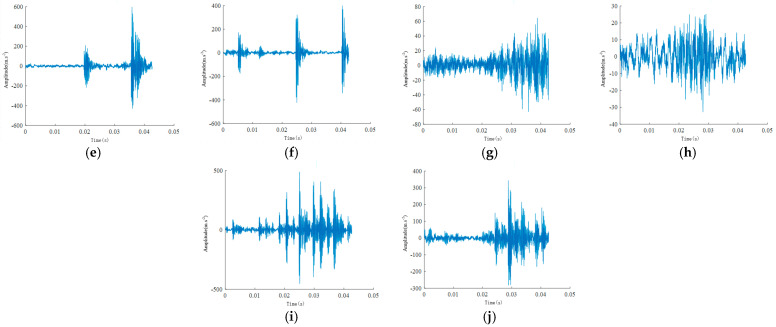
Vibration signal time–domain waveforms under different states: (**a**,**b**) broken tooth, (**c**,**d**) normal, (**e**,**f**) missing tooth, (**g**,**h**) cracked tooth, (**i**,**j**) worn tooth. Odd labels indicate disassembly, and even labels indicate installation conditions.

Figure 12 below shows the time-domain waveforms of different health states collected under the disassembled and installed conditions, with a speed condition of 20 Hz.

**Figure 13:**
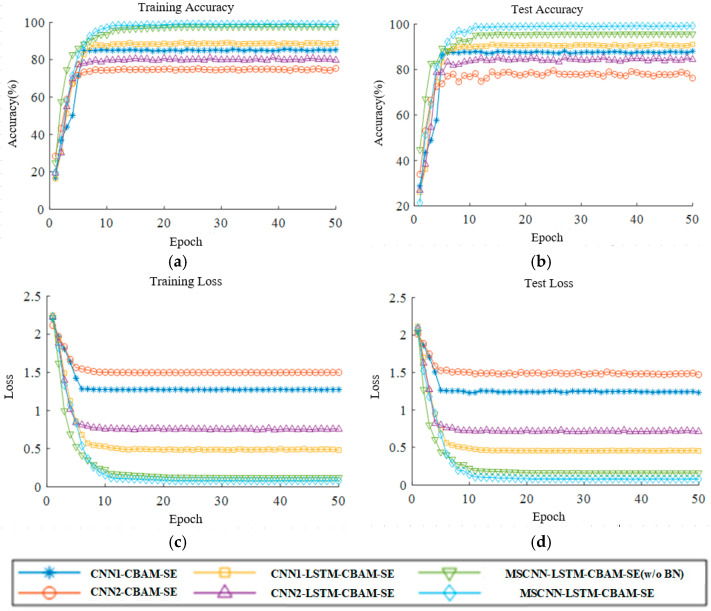
Accuracy and loss curves of Experiment 1: (**a**) training accuracy; (**b**) test accuracy; (**c**) training loss; (**d**) test loss.

This paper evaluates convergence performance through the accuracy curves and loss variation curves of both the training and testing sets. Figure 13 illustrates the training accuracy curves, testing accuracy curves, training loss curves, and testing loss curves for six different methods at a speed of 20 Hz.

Analyzing the experimental results in Figure 13a,b, the MSCNN-LSTM-CBAM-SE (w/o BN) model exhibits a faster convergence rate in the early stages of convergence, compared to the other five fault diagnosis models. However, the MSCNN-LSTM-CBAM-SE model can achieve the highest accuracy, and in the later stages of convergence, its accuracy curve remains stable and does not fluctuate. The accuracy of the other five models generally shows an upward trend in the early stages of convergence, but in the later stages, the models display fluctuations, especially noticeable in the accuracy fluctuations within the testing set. It can be concluded that the MSCNN-LSTM-CBAM-SE network model is capable of achieving higher accuracy in the fault classification of complex vibration signals.

From the analysis of the training loss curves of each model in Figure 13c,d, the MSCNN-LSTM-CBAM-SE model’s loss rate decreases rapidly during the pre-training period, and the loss value can reach a very small value and stabilize in the later stages of iteration. The training loss values of the CNN1-CBAM-SE, CNN2-CBAM-SE, CNN1-LSTM-CBAM-SE, and CNN2-LSTM-CBAM-SE models are higher, and there are fluctuations in the loss values in the later stages of iteration. The loss value of the MSCNN-LSTM-CBAM-SE model is smaller than that of the MSCNN-LSTM-CBAM-SE (w/o BN) model. It can be concluded that the MSCNN-LSTM-CBAM-SE network model can achieve smaller loss values in the fault classification of complex vibration signals.

**Figure 14:**
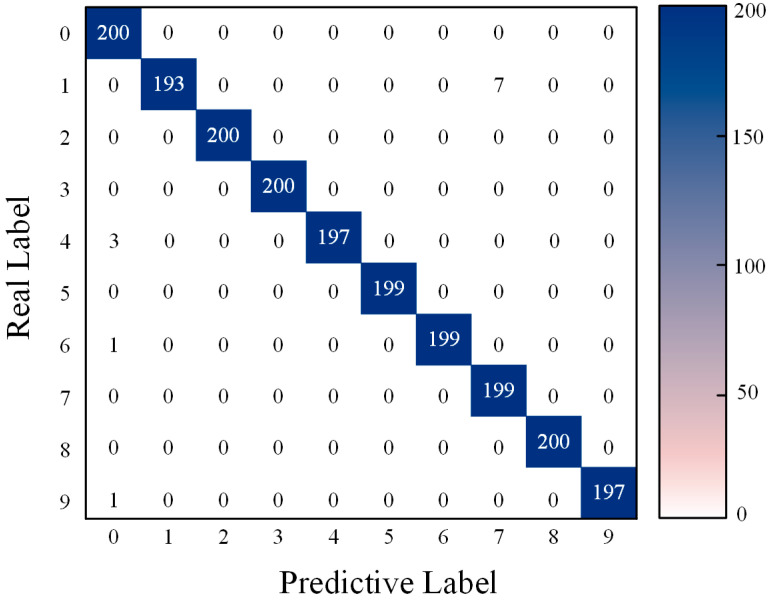
Confusion matrix for MSCNN-LSTM-CBAM-SE fault classification in Experiment 1.

**Figure 15:**
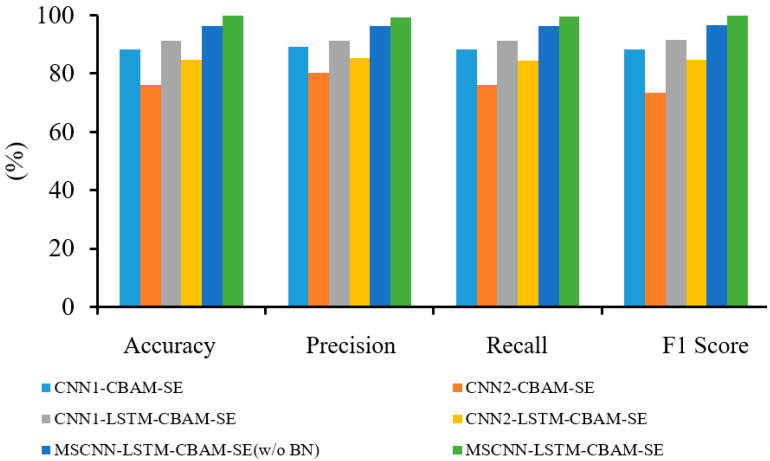
Diagnostic results of six methods in Experiment 1.

Figure 14 displays the confusion matrix obtained by the MSCNN-LSTM-CBAM-SE for gearbox fault classification. Figure 15 presents the average accuracy, precision, recall, and F1 score of six fault diagnosis methods after 30 experiments. The experiments show that CNN1-LSTM-CBAM-SE improves upon CNN1-CBAM-SE by 3.20%, 2.11%, 2.97%, and 2.54% in accuracy, precision, recall, and F1 score, respectively. Similarly, CNN2-LSTM-CBAM-SE outperforms CNN2-CBAM-SE by 8.39%, 5.28%, 8.35%, and 11.61% in these four metrics. Likewise, MSCNN-LSTM-CBAM-SE shows an improvement over MSCNN-LSTM-CBAM-SE (w/o BN) by 3.64%, 2.91%, 3.23%, and 3.07% in these metrics. These indicators reveal that the method proposed in this paper demonstrates significantly stronger diagnostic accuracy and stability. It can accurately classify vibration signals under four different conditions in gearbox fault diagnosis, indicating that MSCNN-LSTM-CBAM-SE is an effective fault diagnosis method.

**Figure 16:**
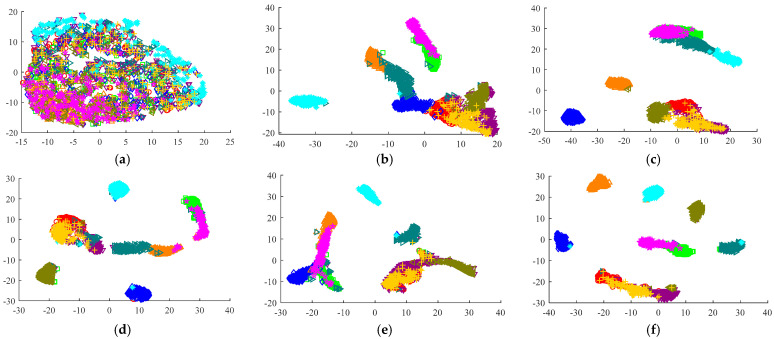

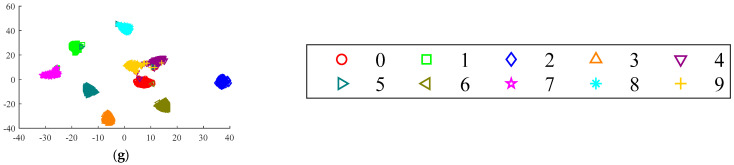
Feature distribution visualization via T–SNE in Experiment 1: (**a**) original test set; (**b**) CNN1–CBAM–SE; (**c**) CNN2–CBAM–SE; (**d**) CNN1–LSTM–CBAM–SE; (**e**) CNN2–LSTM–CBAM–SE; (**f**) MSCNN–LSTM–CBAM–SE (w/o BN); (**g**) MSCNN–LSTM–CBAM–SE.

To further demonstrate the effectiveness of the MSCNN-LSTM-CBAM-SE model, T-SNE is utilized for feature visualization to compare the effects after inputting data into each model for training. Figure 16 shows the original feature distribution of the test set and the final feature visualization distribution of six different fault diagnosis methods. It is evident that the features of the original data are chaotic and overlapping, making it impossible to distinguish among the ten states. However, the trained data reveal that the MSCNN-LSTM-CBAM-SE model yields the best classification results among the six diagrams, effectively proving the MSCNN-LSTM-CBAM-SE model’s highly efficient classification performance.

### 4.3. Experiment 2: HUST Gearbox Fault Diagnosis

#### 4.3.1. Experimental Data Description

**Figure 17:**
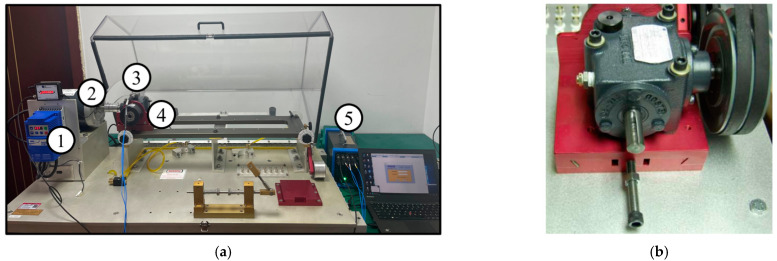
(**a**) Experimental setup for the HUST gearbox dataset: ① speed control; ② motor; ③ acceleration sensor; ④ gearbox; ⑤ data acquisition board. (**b**) Images of the gearbox.

**Figure 18:**
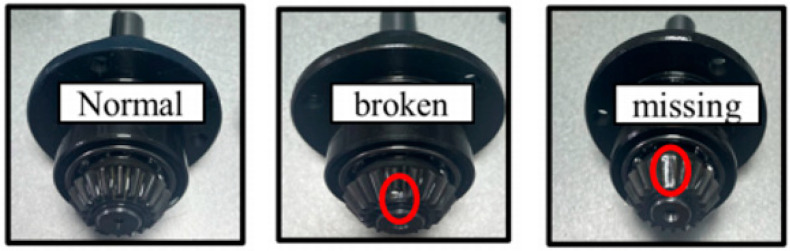
Photographs of the faulty gears.

As shown in Figure 17a, the experimental test rig for data collection consisted of, from left to right, a speed controller, motor, accelerometer, gearbox, and data acquisition board. This test rig utilized a Spectra Quest Machinery Fault Simulator to simulate gearbox fault conditions. The sampling frequency was set to 25.6 kHz, and each data sample recorded 262,144 data points (equivalent to 10.2 s) [39]. This dataset includes the vibration signals of the gearbox under three different health conditions (normal, tooth breakage, and tooth missing) and four different operating conditions. The gearbox used in the experiment is shown in Figure 17b, and the gear conditions (normal, tooth breakage, and tooth missing) are illustrated in Figure 18. All faults were artificially introduced. The experimental data were collected under four different operating conditions, as detailed in Table 5. The specific information on the tested gearbox is provided in Table 6.

According to the information provided in reference [39], the dataset was collected under different operating conditions (cross-working conditions) and across multiple machines (cross-machine scenarios). The dataset includes vibration signals representing various health conditions of the gearbox and considers different operating speeds. The data were collected under steady-state conditions.

#### 4.3.2. Experimental Results and Analysis

The dataset comprises three health states: broken tooth, normal, and missing tooth. In the experiment, 2048 data points in the X direction were selected as a dataset for a single sample. For each health state, data files with an operational condition of 20 Hz rotational speed and a load of 0.113 Nm were chosen, with each file containing 2048 data points from the X-direction sensor as a dataset for a single sample. For each fault type, 1000 samples were extracted, and they were divided into training and testing sets at an 8:2 ratio, meaning that for each fault, the training set contained 800 samples, and the testing set contained 200 samples. The specific details are shown in Table 7 below.

**Figure 19:**
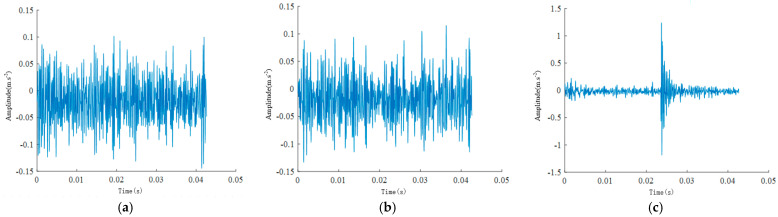
Vibration signal time–domain waveforms for (**a**) broken tooth, (**b**) normal, and (**c**) missing tooth states.

Figure 19 below presents the time-domain waveforms collected under different health states at an operational condition of 20 Hz.

**Figure 20:**
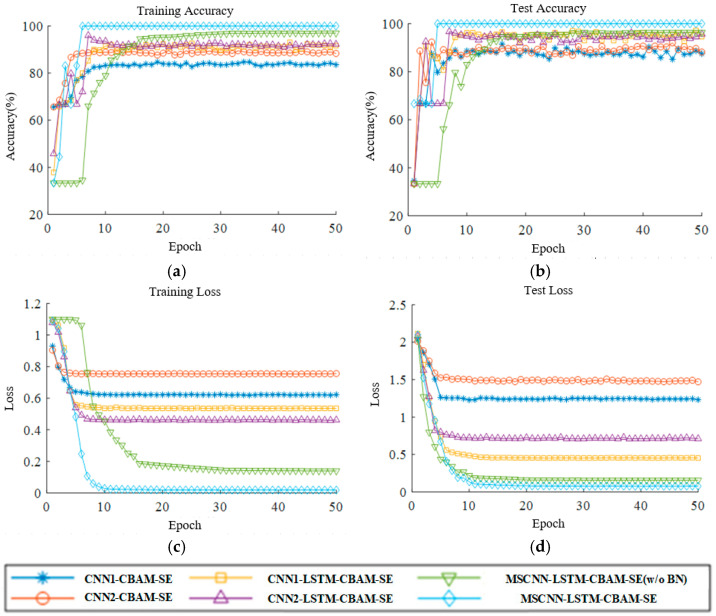
Accuracy and loss curves of Experiment 2: (**a**) training accuracy; (**b**) test accuracy; (**c**) training loss; (**d**) test loss.

This paper assesses convergence performance through the accuracy and loss variation curves for both the training and testing sets. Figure 20 presents the training accuracy curves, training loss curves, testing accuracy curves, and testing loss curves for six different methods at a frequency of 20 Hz.

Analyzing the experimental results in Figure 20a,b, it is observed that the MSCNN-LSTM-CBAM-SE model, despite experiencing some fluctuations in accuracy during the initial rounds of training and testing, is the first to reach the highest accuracy and maintains stability thereafter. The CNN2-LSTM-CBAM-SE and MSCNN-LSTM-CBAM-SE (w/o BN) models exhibit a phenomenon of not increasing in accuracy in the early stages. The CNN1-CBAM-SE, CNN2-CBAM-SE, CNN1-LSTM-CBAM-SE, CNN2-LSTM-CBAM-SE, and MSCNN-LSTM-CBAM-SE (w/o BN) models generally show an upward trend in the pre-convergence phase, but exhibit fluctuations in the later stages of convergence, especially noticeable in the testing accuracy graphs. It can be concluded that the MSCNN-LSTM-CBAM-SE network model is capable of achieving high accuracy in the fault classification of complex vibration signals.

From the analysis of the training loss curves for each model in Figure 20c,d, the MSCNN-LSTM-CBAM-SE model’s loss rate decreases rapidly during the pre-training period, reaching a very small value and stabilizing in the later stages of iteration. The CNN2-CBAM-SE model exhibits the highest loss value, approaching 0.8, whereas the MSCNN-LSTM-CBAM-SE (w/o BN) shows a slower decrease in the loss value, only demonstrating convergence after 16 iterations. The training loss values for the CNN1-CBAM-SE, CNN2-CBAM-SE, CNN1-LSTM-CBAM-SE, and CNN2-LSTM-CBAM-SE models are significantly higher, all above 0.5. It can be concluded that the MSCNN-LSTM-CBAM-SE network model is capable of achieving smaller loss values in the fault classification of complex vibration signals.

**Figure 21:**
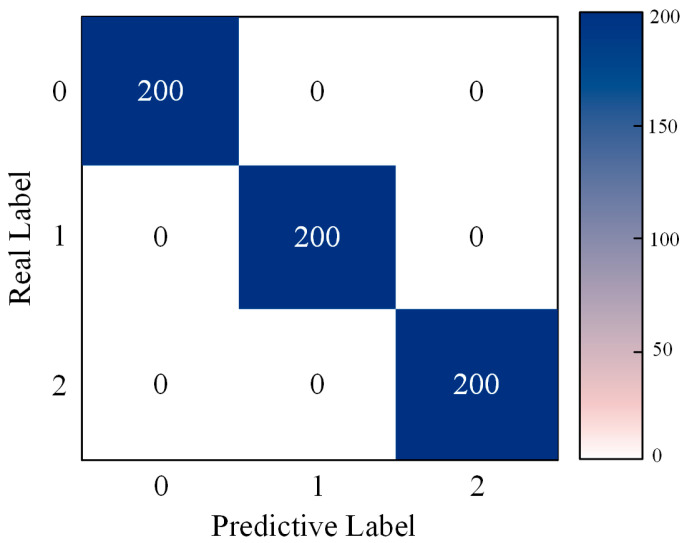
Confusion matrix for MSCNN-LSTM-CBAM-SE fault classification in Experiment 2.

**Figure 22:**
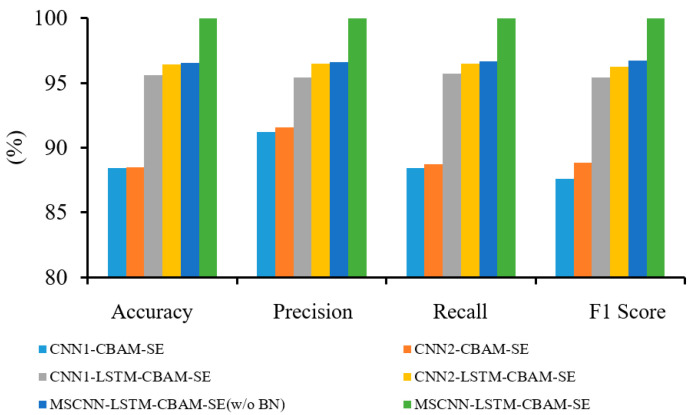
Diagnostic results of six methods in Experiment 2.

Figure 21 presents the confusion matrix obtained for gearbox fault diagnosis, using the method proposed in this paper on the dataset. Additionally, the evaluation of the aforementioned models was conducted using the metrics defined in Equations (13)–(16), with the experimental results shown in Figure 22.

As shown in Figure 22, the MSCNN-LSTM-CBAM-SE model achieved 3.31%, 3.16%, 3.17%, and 3.16% improvements in accuracy, precision, recall, and F1 score, respectively, compared to the MSCNN-LSTM-CBAM-SE (w/o BN) model. Similarly, the CNN1-LSTM-CBAM-SE model outperformed the CNN1-CBAM-SE model by 7.21%, 4.18%, 7.32%, and 7.99% in these four evaluation metrics, while the CNN2-LSTM-CBAM-SE model surpassed the CNN2-CBAM-SE model by 7.98%, 4.97%, 7.72%, and 7.64%, respectively. In summary, the six experimental results demonstrate that the MSCNN-LSTM-CBAM-SE model can more effectively achieve gear fault recognition, providing key technical support for the intelligent fault diagnosis of gearboxes.

**Figure 23:**
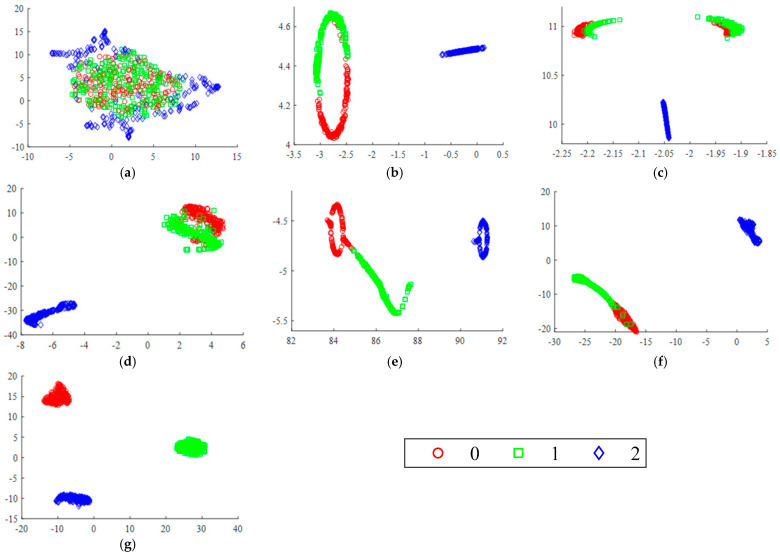
Feature distribution visualization via T–SNE in Experiment 2: (**a**) original test set; (**b**) CNN1–CBAM–SE; (**c**) CNN2–CBAM–SE; (**d**) CNN1–LSTM–CBAM–SE; (**e**) CNN2–LSTM–CBAM–SE; (**f**) MSCNN–LSTM–CBAM–SE (w/o BN); (**g**) MSCNN–LSTM–CBAM–SE.

To further demonstrate the effectiveness of the MSCNN-LSTM-CBAM-SE model, T-SNE was used for feature visualization to compare the effects of data input into each model after training. Figure 23 shows the original feature distribution of the test set and the final feature visualization distribution after classification by six different fault diagnosis methods. It is evident that the features of the original data are mixed and overlapping, making it difficult to distinguish among the ten states. In contrast, the data trained with the MSCNN-LSTM-CBAM-SE model exhibit clear classification effects. From the six comparative diagrams, it is clear that this model performs best in classification tasks, powerfully proving its excellent classification capability.

### 4.4. Additional Experiments

**Figure 24:**
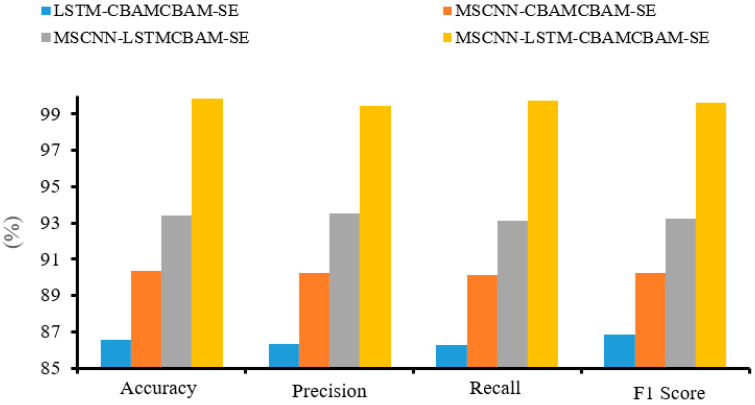
Ablation study comparison chart.

Building upon the two experiments above, this paper further conducts ablation studies to investigate the contribution of each module. As shown in Figure 24, the experiments maintain consistent parameters and utilize the same two datasets as before, with only the inclusion or exclusion of certain modules. The final results were obtained by averaging the outcomes of two trials. Additionally, this paper compares the performance of the MSCNN-LSTM-CBAM model against the other models presented in Table 8. The experiments employed the same datasets and parameter settings, and the results indicate that, while the other methods also perform well, the model proposed in this paper exhibits a slightly better performance.

Based on the analysis of the experimental results, the following conclusions can be drawn:

(1) As shown in Figure 13 and Figure 20, the accuracy curve and loss curve of the MSCNN-LSTM-CBAM-SE model converge after 10 training iterations. This indicates that the MSCNN-LSTM-CBAM-SE model is able to effectively extract fault-related vibration signals from the gearbox vibration data, enabling accurate fault classification.
(2) The results from Experiments 1 and 2 show that the four evaluation metrics of the MSCNN-LSTM-CBAM-SE model are higher than those of the MSCNN-LSTM-CBAM-SE (w/o BN) model. This suggests that the introduction of Batch Normalization (BN) layers in the convolutional neural network can enhance the feature representation and extraction capabilities of the network. The BN layers normalize the internal covariate shift, which helps to improve the stability and convergence speed of network training, and may also enhance the model’s generalization ability.
(3) As shown in Figure 24, the classification accuracy of the proposed model is over 99%, which is higher than that of the other three models without the proposed modules. Furthermore, when compared to the five other models in Table 8, the proposed model demonstrates improved accuracy and a lower standard deviation, further proving its advantage in classification accuracy.

In summary, the MSCNN-LSTM-CBAM-SE model outperforms other methods in all evaluation metrics, including accuracy, precision, recall, and F1 score. This indicates that the proposed model can effectively perform gear fault classification under constant operating conditions, exhibiting high diagnostic performance and practical value.

## 5. Conclusions

This paper proposes a new intelligent fault diagnosis method for gearboxes—the MSCNN-LSTM-CBAM-SE model. The core innovation of this method lies in the introduction of the CBAM-SE module, which significantly enhances the model’s ability to capture critical fault features by combining CBAM and SE techniques. The MSCNN-LSTM-CBAM-SE model not only utilizes the feature extraction capability of the multi-scale convolutional neural network, but also strengthens the feature representation through the CBAM-SE module, while maintaining the LSTM layer’s sensitivity to temporal information. The effectiveness of this method was validated using the WT-Planetary gearbox dataset and the 2024 HUST gearbox dataset. In Experiment 1, the accuracy of MSCNN-LSTM-CBAM-SE was 99.81%. In Experiment 2, the accuracy was 99.89%. In both experiments, the proposed method effectively diagnosed gearbox faults and demonstrated more competitive fault diagnosis performance compared to other models. The gearbox fault diagnosis model proposed in this paper is primarily used for severe gearbox faults and faults that occur during installation and disassembly. However, the experiments conducted in this study were performed on the currently available public datasets, and may do not fully represent real-world mechanical fault diagnosis. Additionally, the experiments were conducted under stable operating conditions, while real-world industrial environments are subject to constant changes, so the proposed model requires further testing in actual environments.

Future research plans: (1) obtain real-world fault data from mechanical equipment to conduct further validation and research; (2) verify whether the proposed model can accurately identify various faults under varying operating conditions.

## Figures and Tables

**Table 1:** Relationship between predicted values and true labels.

		Predicted Values	
		Positive	Negative
True Values	Positive	True Positive (TP)	False Negative (FN)
	Negative	False Positive (FP)	True Negative (TN)

**Table 2:** Parameter list for the MSCNN-LSTM-CBAM-SE fault diagnosis model.

Layer Structure	Convolutional Kernel Size (Number)	Stride	Input Size	Output Size	Number of Parameters
Conv_1-BN-ReLU	6 × 1(50)	4	[1, 2048]	[50, 512]	350
Conv_2-BN-ReLU	6 × 1(40)	1	[50, 512]	[40, 507]	12040
Pooling_1	2 × 1	2	[40, 507]	[40, 254]	0
Conv_3-BN-ReLU	6 × 1(30)	1	[40, 254]	[30, 249]	7230
Conv_4-BN-ReLU	6 × 1(30)	2	[30, 249]	[30, 123]	5430
Pooling_2	2 × 1	2	[30, 123]	[30, 62]	0
Conv_5-BN-ReLU	20 × 1(50)	4	[1, 2048]	[50, 510]	1050
Pooling_3	2 × 1	2	[50, 510]	[50, 256]	0
Conv_6-BN-ReLU	20 × 1(30)	2	[50, 256]	[30, 123]	30030
Pooling_4	2 × 1	2	[30, 123]	[30, 62]	0
feature fusion	-	-	[30, 62]	[30, 62]	0
			[30, 62]		
LSTM1	-	-	[30, 62]	[30, 60]	21600
LSTM2	-	-	[30, 60]	[30, 60]	28800
CBAM-SE	-	-	[30, 60]	[30, 60]	6660
Fc	-	-	[30, 60]	[30, 10]	610

**Table 3:** Parameters of the planetary gearbox.

Tooth number	Ring gear	100
	Sun gear	28
	Planet gear(number)	36(4)
Sun Gear Failure Frequency	(25/8)*f_r_*	
Tooth Mesh Frequency	(175/8)*f_r_*	

Note: *fr* denotes the rotational frequency of the sun gear.

**Table 4:** WT-Planetary gearbox experimental dataset.

Label	Device Status	Sample Type	Number of Training Set Samples	Number of Testing Set Samples
0	disassembly	Broken tooth	800	200
1	installation	Broken tooth	800	200
2	disassembly	Health	800	200
3	installation	Health	800	200
4	disassembly	Missing tooth	800	200
5	installation	Missing tooth	800	200
6	disassembly	Cracked tooth	800	200
7	installation	Cracked tooth	800	200
8	disassembly	Worn tooth	800	200
9	installation	Worn tooth	800	200

**Table 5:** Operational conditions table.

Frequency (Hz)	Load (Nm)
20	0.113
25	0.226
30	0.339
35	0.452

**Table 6:** Detailed parameter table of the gearbox.

Ratio	1.5: 1	Gearbox Model	Hub City M2
Pitch angle pinion	33°41′	Pitch angle gear	56°19′
Pitch diameter pinion	1.125 inches	Backlash tolerance	0.001–0.005 inches
Pitch diameter gear	1.6875 inches	Number teeth pinion	18
Number teeth gear	27	Gear bearing	2 bearings
Pinion bearing	NSK 6202 (1 bearing)	Pressure angle for gear and pinion	20°

**Table 7:** HUST gearbox experimental dataset.

Label	Sample Type	Number of Training Set Samples	Number of Testing Set Samples
0	broken tooth	800	200
1	Normal	800	200
2	missing tooth	800	200

**Table 8:** Comparison with alternative techniques.

Methods	Accuracy	Standard Deviation
MSCNN	97.22%	0.438%
BiConvLSTM	97.85%	0.761%
DenseNet121	98.26%	0.143%
MDIFN	98.76%	0.173%
CBAM-ResNeXt50	99.45%	0.146%
MSCNN-LSTM-CBAM-SE	99.85%	0.114%

## Data Availability

The WT-Planetary gearbox dataset used in experiment 1 is accessible at: https://github.com/Liudd-BJUT/WT-planetary-gearbox-dataset (accessed on 13 February 2024). The HUST gearbox dataset used in experiment 2 is accessible at: https://github.com/CHAOZHAO-1/HUSTgearbox-dataset (accessed on 1 February 2024).
